# A Cross‐Sectional Study Exploring the Suitability of Skin Hydration Measurement Devices for Use on the Foot

**DOI:** 10.1002/jfa2.70104

**Published:** 2025-11-28

**Authors:** Jennifer Andrews, Chris Nester, Carina Price, Farina Hashmi

**Affiliations:** ^1^ The School of Health and Society The University of Salford Salford UK; ^2^ School of Allied Health Professions and Pharmacy Keele University Keele UK

**Keywords:** callus, corns, dermatology, plantar skin, podiatry, quantification, technology, xerosis

## Abstract

**Introduction:**

Foot skin xerosis is common, particularly in older people and people with diabetes. Efficacy of emollient treatment of xerosis can be measured using skin hydration measurement devices. None of the devices currently available, however, have been explicitly assessed for their suitability for use on the skin of the foot. The plantar skin has a morphology and composition disparate from non‐plantar skin sites, with a stratum corneum (SC) 16 times thicker than non‐plantar skin SC. The shallow measurement depth of hydration measurement devices (0.015 mm for the Corneometer CM825) could be collecting data from incommensurate locations within plantar and non‐plantar skin. The aim of this study is to examine how data collected using three hydration measurement devices with different measurement depths (Corneometer CM825, MoistureMeter D and MoistureMeter SC) correlate with tissue characteristics known to vary with skin hydration (hardness, elasticity, surface texture and patient perception) to inform their future use.

**Methods:**

Individuals aged 20–40 were recruited to attend the University of Salford Skin laboratory for data‐collection. Following a 15‐min acclimatisation period, measures were taken from four skin sites (plantar and non‐plantar) using three hydration measurement devices, the SATRA STD 226 Durometer (SATRA Technology, Kettering, UK), Dermalab Elasticity probe (Cortex Technology, Hadsund, Denmark), Visioscan VC98 (Courage and Khazaka, Koln, Germany) and the Foot Skin health Questionnaire. Correlation analyses were conducted using SPSS (IBM SPSS Statistics Version 29.0.1.0).

**Results:**

Thirty‐two participants were recruited (mean age ± (SD):27.9 ± 4.8; 53% female). The Corneometer CM825 (*n* = 20) and MoistureMeter SC (*n* = 32) demonstrate consistent weak‐moderate strength correlations with skin elasticity, hardness and texture for both plantar and non‐plantar skin. The MoistureMeter D (*n* = 32), however, correlated stronger with the physical characteristics of plantar skin than non‐plantar skin. The only device that found a statistically significant difference between self‐perceived ‘dry’ or ‘not dry’ skin was the Corneometer CM825 (Mann‐Whitney *U* test *p* = 0.009).

**Conclusion:**

The skin site being measured should guide the selection of a hydration measurement device. Future work should include a similar assessment using low‐cost devices that are accessible to health care practitioners and expansion of the work to include xerotic skin.

**Abbreviations**
D3: dorsum of the third metatarsophalangeal joint
H: heel
MA: medial arch
P3: plantar third metatarsophalangeal joint

## 1 Background

The foot is a common location for skin pathologies such as corns and calluses (hyperkeratosis) which are strongly associated with disabling foot pain and requiring health professional input or self‐management [1]. ∼60% of people 65+ have foot hyperkeratosis [2]. These lesions can cause pain, leading to reduced activity, low mood and increased falls [3, 4] and ultimately reduced health‐related quality of life [5, 6, 7]. For people with impaired vascular or sensory status in their feet (e.g., people with diabetes (∼25% of people 75+)) these are also associated with an increased risk of foot ulceration [8] and subsequently limb loss and mortality [9].

Foot skin water content determines its physical characteristics and mechanical behaviour: dry skin is hard, inelastic and has a rough surface texture with consequences for friction. Corns and callus, as well as other foot skin pathologies, are associated with xerosis (or dryness) [10]. Consistent with the importance of water, the pathogenesis of corns and callus and many other foot skin pathologies, includes xerosis [10] and assessment of skin hydration is central to understanding and thereafter preventing or treating, these common but important skin pathologies.

Devices to measure skin hydration, such as the Corneometer CM825 (Courage and Khazaka, Colne, Germany), MoistureMeter D and MoistureMeter SC (Delfin Technologies, Kuopio, Finland), are largely designed for cosmetic applications [11]. This is important because plantar foot skin is significantly different in form and function than cosmetic sites. Most notably the stratum corneum (SC) is 16 times thicker in plantar compared to non‐plantar sites [12]. This is important because most devices evaluate water content at fixed distances from the skin surface. This is 0.015 mm for the most commonly reported device, Corneometer CM825 [11, 13], whose measures will relate to the far more superficial SC in plantar foot skin than non‐plantar skin [12] (Table 1). The same measurement devices therefore measure hydration in different anatomical skin layers, where cell form and tissue function are different and hydration levels should vary. This renders data comparison complex, perhaps erroneous or even misleading and could lead to incorrect conclusions relating to skin health and mechanical skin behaviour. This is compounded by the fact that skin pathologies often involve thickening of the SC layer so that the depth at which hydration is measured can vary in unquantifiable ways.

**TABLE 1:** Measurement depth of devices and corresponding tissues targeted in plantar and dorsal foot skin.

Device	Measurement depth (mm)	Skin structure at measurement depth	
		Plantar skin	Dorsal skin
Corneometer CM825	0.015	Superficial 3% of SC	Superficial 42% of SC
MoistureMeter D	500, 1500, 2500 (probe dependant)	500 mm: Viable epidermis	Subcutaneous tissues
		1500 and 2500 mm: Subcutaneous tissues	
MoistureMeter SC	Not given	N/A	N/A

*Note:* SC thickness data extracted from Vela‐Romera et al. [14].

Alternatives to the Corneometer CM825 measure skin hydration using different mechanisms or at different depths that potentially make them more suitable for use on the plantar skin. For example, the MoistureMeter D utilises tissue dielectric constant to quantify tissue water content at measurement depths of 0.5, 1.5, 2.5 and 4 mm [15] and the MoistureMeter SC uses capacitance but reports a measure of ‘effective hydration’, that takes both dryness and thickness of the outermost skin layer into account [16]. To date, measures taken using these alternatives have not been compared to Corneometer CM825.

Self‐management of skin pathology (or accessing professional care) is triggered by an individual's assessment, interpretation and experience of skin condition. Indeed, skin pathology is commonly treated using over the counter products that rely on visible or perceivable changes in skin properties [17], including skin texture that relates to hydration. The relationship between skin hydration and a person's experience of foot skin is therefore important but also complex to understand if measures of hydration are invalid or difficult to interpret. Previous studies demonstrated agreement between skin hydration derived using the Corneometer CM825 and a clinician's perception of skin pathology, but have not investigated perceptions of people experiencing the foot pathology [10]. If the various ways to measure skin hydration are impacted differently by the skin pathology being experienced by a person (e.g., due to changes in skin thickness or hardness), then any apparent agreement between measured and persons' perceived foot skin hydration may be likewise invalid. This has implications for how changes in skin hydration due to a topical skin product (or other approach) measured in experimental research are experienced by individuals and subsequently their self‐management behaviours.

The water content of skin impacts its physical characteristics: dry skin is hard, inelastic and has a rough surface texture [10]. In this study, several devices are used to quantify these characteristics. With the absence of a gold standard measure of water content of foot skin for comparison, the relationship between the hydration data collected and physical characteristics can be explored [10]. The association of outcome measures from each device and these physical skin characteristics indicates the device's ability to detect meaningful hydration—that is data that has a tangible link to skin health or an individual's propensity to seek out xerosis treatment. A strong agreement with skin hardness could support a device's use to monitor change in foot skin following the application of a callus‐reducing emollient for example. This improved understanding of how foot skin hydration should and can be measured with commercially available devices will enhance the quality of foot‐emollient research, supporting innovation in the sector and ultimately facilitating better management of dry foot skin conditions and poor health‐related quality of life associated with these.

The purpose of this study is to assess the strength of the relationships between the three hydration measurement devices and the physical characteristics of the skin and participant perception of skin health. These data contribute to a discussion around the acceptability of these devices for use on the foot skin: described herein as the ‘suitability’ of the device for use on the foot.

## 2 Methods

The study protocol was reviewed and approved by The University of Salford Ethics Panel reference number: 3137. Each participant completed a written informed consent form prior to taking part in this study.

### 2.1 Participants

Able bodied people aged 20–40 years of age were recruited to attend a single data collection session at The University of Salford, Manchester, UK between April and May 2022. Participants were excluded from the study if they had diabetes or a current foot skin pathology (excluding mild dry or callused skin). Exclusion criteria were selected to reduce data variability, improving the strength of the statistical testing presented and contributing to the purpose of this work; to assess the functionality of the devices being used.

### 2.2 Protocol

Participants were asked to refrain from applying emollient to their feet for 7 days prior to data‐collection. Participants were seated on a plinth with their feet raised and uncovered for a fifteen‐minute acclimatisation period. As humidity and temperature impact skin hydration measures [18, 19], environmental conditions within the laboratory were recorded at the commencement and close of each data‐collection session and assessed for variability between participants.

#### 2.2.1 Calibration

The Corneometer CM825 and MoistureMeter SC were calibrated prior to data‐collection beginning and the MoistureMeter D was calibrated through an internal mechanism prior to each data‐collection session.

**FIGURE 1:**
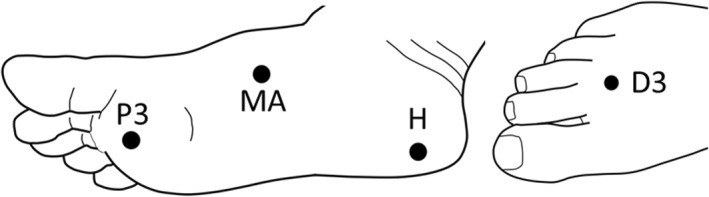
Diagrammatic representation of foot skin testing locations. D3, dorsum of the third metatarsophalangeal joint; H, heel; MA, medial arch; P3, plantar aspect of base of the first metatarsal.

Six devices were used to collect data on the foot skin hydration and physical behaviour. The order of use was determined by their perceived influence on other measurements (i.e. least likely to influence other measurements to most): Corneometer CM825, MoistureMeter SC, MoistureMeter D (hydration), SATRA STD 226 Digital Durometer (hardness), Dermalab Elasticity probe (elasticity), Visioscan VC98 (surface texture (contrast parameter of output indicating the roughness, scaliness etc. of the skin surface)). Each device was used to collect data from each skin site, working from those most proximal to the most distal (Figure 1; D3, H, MA, P3) and alternating between left and right body areas. Each hydration measurement device was applied to each skin site three times and the mean value of these used for analysis. The SATRA STD 226 Digital Durometer, Dermalab Elasticity probe and Visioscan VC98 were each applied once. Skin elasticity is represented by the maximum skin distension (in mm) in a 0.5 s period of 400 mbar negative pressure and given in units mm/bar, as per the work conducted by Hashmi et al. [10].

#### 2.2.2 Skin Measurement Sites

Skin hydration and physical characteristics data were collected at four locations: the dorsum of the third metatarsophalangeal joint (D3), 2 cm inwards from the posterior centre of the heel (H), the plantar aspect of base of the first metatarsal (medial arch (MA)), plantar third metatarsophalangeal joint (P3) (Figure 1). These sites were identified through palpation by the researcher (a Podiatrist (JA)) and a mark made with a skin‐safe pen 1 cm lateral to each site to guide device placement.

Participants were asked to complete a survey to identify whether they perceived their foot skin to be ‘dry’ (yes/no) at the same four measurement sites.

### 2.3 Statistical Analysis

Statistical Package for the Social Sciences (SPSS) 20 software (IBM, USA) was used for all data analysis. Data were assessed for normality of distribution through use of the Shapiro‐Wilks test and review of Q‐Q plots, measures of central tendency and histograms to define data as parametric or non‐parametric.

Pearson's correlation coefficient and Spearman's rank correlation coefficient (parametric or non‐parametric, respectively) were used to determine the strength of relationship between data on skin hydration and physical characteristics. Interpretation of the strength of correlations was based on advice for medical research [16]. Participant indication of skin dryness (yes/no) were treated as groups ‘dry’ and ‘not dry’ respectively and hydration data (mean of all anatomical sites) between groups were compared using the Student's *t*‐test and Mann‐Whitney *U* test (parametric and non‐parametric). The Bonferroni correction was applied to reduce the risk of a type 1 error arising as a result of the multiple comparisons undertaken within this analysis generating a threshold *p*‐value of ≤ 0.01 for statistical significance [20]. No outliers were identified although several high hydration values were identified; these were not removed due to consistency with other skin sites in the same individual, indicating that these were representative of normal variation.

## 3 Results

Thirty‐two participants were recruited (Table 2). Environmental conditions remained stable throughout data‐collection appointments (Table 2). Fewer participants were tested using the Corneometer CM825 (20) than the other devices (32) due to device malfunction during the data‐collection period. The results below represent the full dataset for each device.

**TABLE 2:** Participant demographics and environmental conditions.

*N*	Age (years) (SD)	% female [*n*]	Mean start temperature (°C) (SD)	Mean end temperature (°C) (SD)	Mean temperature fluctuation (°C) (SD)	Mean start RH (%) (SD)	Mean end RH (%) (SD)	Mean RH fluctuation (%) (SD)
32	27.9 (4.8)	53% [20]	20.4 (1.3)	20.4 (1.6)	0.04 (1.17)	41.5 (6.6)	40.8 (7.3)	0.72 (2.73)

Abbreviation: RH, room humidity.

The Corneometer CM825 and MoistureMeter SC both demonstrate weak‐moderate correlation with skin elasticity, hardness and texture at most foot skin sites (Table 3). The strongest correlations are for the Corneometer CM825 with skin hardness (0.132 to −0.633 (Table 3)) and the MoistureMeter SC hydration with skin texture (−0.160 to −0.682 (Table 3)).

**TABLE 3:** Spearman's rank analysis for hydration measurement devices (listed in the lefthand column) and each skin characteristic (A: Elasticity, B: Hardness and C: Texture) across four left and four right foot sites.

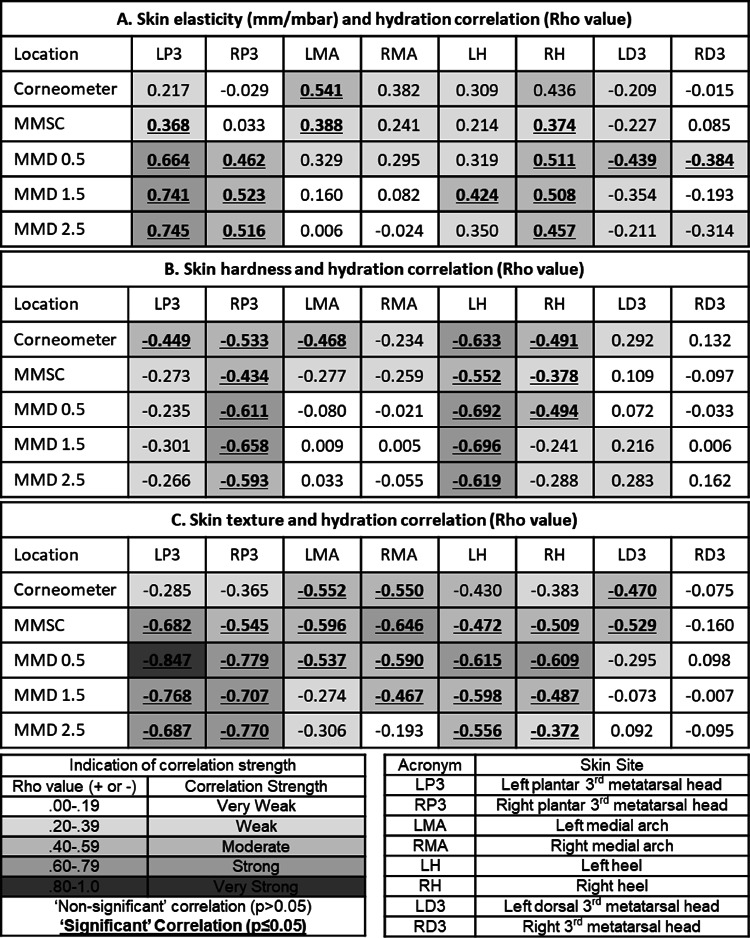

*Note:* Corneometer CM825 *n* = 20, all other devices *n* = 32.

Data collected using three different probes for the MoistureMeter D correlated with skin elasticity, hardness and texture to varying degrees depending upon the skin site (Table 3). The MoistureMeter D 1.5 and 2.5 probes consistently correlate stronger with the physical characteristics of plantar skin (1.5 range: 0.741 to −0.768, 2.5 range: 0.745 to −0.770) than non‐plantar skin (1.5 range: 0.216 to −0.354, 2.5 range: 0.283 to −0.314) and to a lesser degree, the medial arch skin (1.5 range: 0.160 to −0.467, 2.5: 0.055 to −0.306) (Table 3).

This same pattern is demonstrated by the MoistureMeter D 0.5 probe data with skin hardness (plantar sites: −0.235 to −0.692, medial arch: −0.021 to −0.080, dorsal skin: 0.072 to −0.033 (Table 3)) but not skin texture (all plantar and medial arch sites: −0.537 to −0.847). The MoistureMeter D 0.5 probe demonstrates a weak‐strong positive correlation with skin elasticity across all skin sites (0.664–0.295 (Table 3)).

**FIGURE 2:**
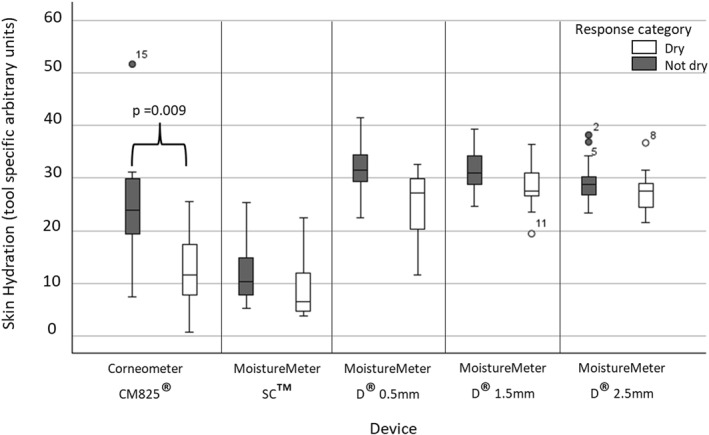
Skin hydration as measured using the five devices for groups of individuals who responded ‘yes’ (white) or ‘no’ (grey) to the question: Is your foot skin dry?. Statistically significant (*p*‐value = < 0.01) results of a Mann‐Whitney *U* test between groups are indicated. Outlier data are indicated by a circle and participant number. Corneometer CM825 *n* = 20, all other devices *n* = 32.

When comparing skin hydration for individuals who perceive their foot skin as dry or not dry for all devices, individuals who perceived their foot skin to be dry had lower hydration values (mean of anatomical sites on the right ride of the body), which was statistically significant (*p*‐value = 0.009) in the data collected using the Corneometer CM825 (Figure 2).

## 4 Discussion

In this study, very weak to very strong correlations were calculated between skin hydration and physical characteristics at different foot sites for a range of measurement devices. Similarly, participant perception of their skin dryness in relation to hydration measurements using different devices varied. Data collected using the Corneometer CM825 demonstrated a significant difference between participant‐reported ‘dry’ and ‘not’ dry skin categories. For this reason, skin sites and skin characteristics of interest are key factors that should influence selection of skin hydration measurement devices.

The three probes of the MoistureMeter D measure tissue hydration at different measurements depths [15]. Stronger correlations were demonstrated by the deeper penetrating probes (1.5 and 2.5 mm) at thicker plantar skin sites (heel and plantar third metatarsal head), compared to weaker correlations at areas with thin skin (medial arch and the foot dorsum). This is not reflected to the same degree with the 0.5 mm probe for skin elasticity or surface texture.

Applying knowledge of tissue morphology across these sites can explain this: when applied to the non‐plantar skin the 0.5 mm probe is collecting data from the SC, the viable epidermis and beyond [21] whereas on the plantar skin this is collecting data from the SC alone [22]. Similarly, the measurement region of the 1.5 and 2.5 mm probes contain a much higher portion of the dermis on the non‐plantar skin sites than on plantar skin sites. The water content of the dermis would not be expected to influence skin behaviour of healthy individuals such as those involved in this study. A deeper measurement depth would therefore reduce the strength of correlations with the physical characteristics of the skin. The thick SC minimises this effect on plantar skin.

The MoistureMeter SC and Corneometer CM825 demonstrate similar patterns of correlation direction and strength with all physical characteristics for most skin sites, that is higher skin hydration is associated with higher elasticity and reduced surface texture and skin hardness. Corneometer CM825 data has a slightly stronger and more consistent correlation (between skin sites) with skin hardness than the MoistureMeter SC and the reverse is true for skin texture. These devices are reported to use the same measurement mechanism, but their data‐collection regions within the skin are described differently.

The stronger correlation demonstrated by the Corneometer CM825 may suggest hardness is influenced more by the most superficial 0.015 mm of tissue, than the characteristics of the whole SC (assessed by the MoistureMeter SC) and the opposite is true for skin texture. Future work should investigate measurement depth of these two capacitance‐based devices in vivo or ex vivo, to establish whether this is the case or if these differences in correlation strength are in fact the result of other confounding factors. One such factor could be the attenuation of the signal emitted by the devices as a result of superficial SC dryness resulting in a tool having a markedly different measurement depth. Simultaneous data collection with a device capable of measuring SC hydration across its depth would facilitate this comparison.

In this study, a positive, albeit inconsistent across sites, correlation is demonstrated between skin hydration and elasticity at all plantar and medial arch skin sites. Hashmi et al. [10] previously reported statistically significant positive correlations between skin hydration and elasticity in the centre and edge of callus (*r*‐values: 0.56 and 0.29) and fissures (*r*‐values: 0.65 and 0.65), xerotic heel skin and the fifth metatarsal base (*r*‐values: 0.34 and 0.25), however not the normal plantar metatarsal area (*r*‐value: 0.13). That the strongest correlations between skin hydration and elasticity were found at hyperkeratotic areas with thickened stratum corneum [23] suggested SC thickness was influential on the impact hydration might have on elasticity. In the current study however, the medial arch skin demonstrated equivalent or stronger correlation between skin hydration and elasticity compared to plantar heel or metatarsal head sites, despite its thinner SC [22], suggesting thickness has no bearing.

These contrasting outcomes may be due to the elasticity measurement devices differing in these studies: The Cutometer 580 MPA used by Hashmi et al. [10] had an aperture of 8 mm diameter whereas the Dermalab Elasticity probe has an aperture of 10 mm diameter. This larger aperture could introduce more influence of the mobility of deeper subcutaneous tissues into the elasticity data collected within the current study. Additionally, Hashmi et al. [10] recorded the maximum displacement of the skin following 30 s of exposure to 500 mbar negative pressure, 60 fold longer extended exposure. Hashmi et al. [10] did this with the aim of observing viscoelastic properties of the plantar tissues as well as the skin. The extended exposure will have introduced creep and reduced the linearity of the association between hydration and the tissue displacement, weakening the *r*‐values generated and therefore limiting comparability with these data in terms of absolute values.

Despite these methodological differences, the contrasting outcomes from the different populations recruited in Hashmi et al. and the current work imply that water content of the SC in hyperkeratotic lesions is more influential to its physical behaviour than in physiologically healthy skin. It would be beneficial in future to examine the relationship between elasticity and hydration across individuals with varying degrees of xerosis and hyperkeratosis, to isolate the confounding effect thickness has on the relationship between hydration and elasticity with consistent measurement approaches.

### 4.1 Hydration and Surface Texture

In the current study negative correlations (range: −0.847 to −0.285) of mixed statistical significance were consistently identified between skin hydration and surface texture on the plantar surface for different devices. This is anticipated as well‐hydrated skin readily sloughs off keratinocytes at the skin surface, creating a smooth surface (less surface texture), however in xerotic skin this process is impaired, leading to skin flakes forming and remaining adhered to the skin surface (increased surface texture) [24]. This outcome suggests that the hydration measures are suitable for use on plantar foot skin if we use surface texture as a threshold.

### 4.2 Skin Hydration and Perception of Skin Dryness

An individual's perception of their foot skin health is an important factor in identifying skin pathology and influencing adherence to an emollient regime [25, 26]. The ability of a hydration measurement device to collect data that is reflective of an individual's perception of their skin health is therefore important for manufacturers of emollients and those seeking to empower patients to identify and remedy foot skin pathology. Comparison between self‐perceived ‘dry’ and ‘not‐dry’ foot skin groups yielded statistically significant results for the Corneometer CM825 only where those who perceived their feet to be dry had lower hydration values. This may be the case as compared to the other hydration measurement devices, the Corneometer CM825 collects data from superficial tissues, more proximal to the observable skin surface.

This is the first work to evaluate how members of the public perceive their foot skin hydration compared to objective measures of skin hydration. Individuals are sensitive to foot skin dryness and data from the Corneometer CM825 will reflect their perceptions. Future work might explore an individual's sensitivity to changes in their foot skin hydration identified through objective measures. Generation of a minimally detectable difference for changes in foot skin hydration that is perceivable by an individual could contribute to emollient efficacy testing.

### 4.3 Limitations

This study would be strengthened through larger participant numbers and greater diversity in ethnicity, age and health status of participants. It is possible that differences in skin structure and composition associated with these factors may influence the measurements collected using these devices and impact their correlation with other tissue characteristics [27, 28]. For example, in xerosis, plaques of dead keratinocytes remain adhered to the skin surface [24]. Measurements collected using devices with a set measurement depth, that is the Corneometer CM825, could collect hydration data entirely from these plaques of cells or have their measures significantly reduced by their presence. This could reduce the strength of the correlation they demonstrate with the physical characteristics of the skin which may be less impacted by superficial skin plaques. Whilst such factors are unexplored in this work, the generalisability of these findings to a wider population is limited.

During this study, the Corneometer CM825 was hindered by malfunctioning. This resulted in a smaller dataset for this device than others, reducing statistical power of analyses. Despite this, the Corneometer CM825 data generated comparable correlations with skin characteristics to other skin hydration measurement devices. It is likely that stronger correlations would have been evident between the Corneometer CM825 data and other skin characteristics, if this dataset was not limited in size.

Additionally, the skin hydration measurement devices used within this study are not exhaustive of the commercially available devices for this purpose. Several very low‐cost devices are available to clinicians that have not yet been evaluated for use on the foot, the validation of which could provide opportunity for clinicians working in low‐resource settings to monitor skin hydration objectively.

### 4.4 Application in Pathology

As described, further study with the inclusion of older people or people with xerosis would enhance the generalisability of the application of these findings. However, the outcome from this study provides valuable guidance for use by the bodies responsible for innovating within the foot skin‐care environment. With the current dearth of evidence supporting their suitability for use on the unique plantar foot skin, which is often the location for painful and disabling pathology [5, 6], use of hydration measurement devices leaves opportunity for misuse and misinterpretation.

These data provide clear demonstration of the use of hydration measurement devices on the foot skin and the implication of the data they generate in relation to skin characteristics frequently of importance in assessment of emollient efficacy (i.e. influence on skin hardness) and acceptability by patients (i.e. perceptible change in skin features). This will ultimately contribute to improved clinical practice through the delivery of emollient products whose efficacy on the foot skin has been proven using tools supported for use through the insight gained through this study.

## 5 Conclusions

Data demonstrate that devices to measure skin hydration identify variation between foot sites that correlate with expected changes in skin physical properties. Variation between devices in performance between foot site and association with physical characteristics mean both these factors must be considered when selecting the most appropriate hydration measurement device.

If data collection entails both the plantar and dorsal skin surfaces, the Corneometer CM825 is the device which generates data most closely linked with skin hardness, the MoistureMeter SC is more representative of skin hydration associated with surface texture and the MoistureMeter D 0.5 mm probe generates data that most consistently correlates with skin elasticity.

Alternatively, if only the plantar tissues are to be tested, the MoistureMeter D is a valid choice for investigations relating to any skin characteristics other than patient perception of skin dryness. For investigations in which patient perception of skin dryness is of interest, irrespective of skin site, the Corneometer CM825 should be used.

Further work is required to explore the consistency of these relationships in xerotic skin and to expand the remit of these suggestions for practical application of these devices to other, low‐cost hydration measurement devices.

## Author Contributions

**Jennifer Andrews:** conceptualization, methodology, data curation, formal analysis, investigation, writing – original draft, writing – review and editing. **Chris Nester:** funding acquisition, conceptualization, methodology, supervision, writing – review and editing. **Carina Price:** conceptualization, methodology, formal analysis, supervision, writing – review and editing. **Farina Hashmi:** conceptualization, methodology, formal analysis, supervision, writing – review and editing.

## Funding

Scholl Wellness sponsored the PhD studentship within which this work was undertaken.

## Ethics Statement

The study protocol was reviewed and approved by The University of Salford Ethics Panel reference number: 3137.

## Consent

Each participant completed a written consent form prior to taking part in this study.

## Conflicts of Interest

The authors declare no conflicts of interest.

## Data Availability

The data collected and analysed within this study are not publicly available but are available from the corresponding author on request.
